# Association between TLR4 (+896A/G and +1196C/T) Polymorphisms and Gastric Cancer Risk: An Updated Meta-Analysis

**DOI:** 10.1371/journal.pone.0109605

**Published:** 2014-10-07

**Authors:** Quan Zhou, Chenchen Wang, Xiaofeng Wang, Xiongyan Wu, Zhenggang Zhu, Bingya Liu, Liping Su

**Affiliations:** Shanghai Key Laboratory of Gastric Neoplasms, Department of Surgery, Shanghai Institute of Digestive Surgery, Ruijin Hospital, Shanghai Jiao Tong University School of Medicine, Shanghai, People's Republic of China; Indiana University School of Medicine, United States of America

## Abstract

**Background:**

Toll-like receptor 4 (TLR4) is a receptor of lipopolysaccharide in the signaling transduction of gastric epithelial cell. It plays a pivotal role in activation of innate immunity and pathogen recognition and thus acts as a modulator in the development and progression of gastric cancer. Growing studies explored the association of polymorphisms in TLR4 with susceptibility to gastric cancer, but the results have remained controversial and conflicting. To investigate the effect of two selected TLR4 (+896A/G and +1196C/T) polymorphisms on gastric cancer, we performed a meta-analysis.

**Methods:**

A comprehensive search was conducted to identify all eligible case-control publications investigating the association between TLR4 polymorphisms and gastric cancer risk. Odds ratios (OR) and corresponding 95% confidence intervals (CI) were used to assess such association.

**Results:**

Up to March 26 2014, 10 published case-control studies from PubMed and EMBase were available, involving a total of 1888 gastric cancer patients and 3433 control subjects. In the overall meta-analyses, a significantly increased gastric cancer risk was detected in TLR4 +896A/G polymorphism (heterozygous model, AG vs. AA: OR = 1.67, 95% CI, 1.39–2.01; additive model, G vs. A: OR = 1.64, 95% CI, 1.37–1.95) and TLR4 +1196C/T polymorphism (heterozygous model, CT vs. CC: OR = 1.42, 95% CI, 1.11–1.81; additive model, T vs. C: OR = 1.36, 95% CI, 1.08–1.72), similar results were obtained in the subgroup analyses of Caucasian, whereas no associations were detected in any genetic models of non-Caucasian.

**Conclusions:**

The overall results suggest that TLR4 polymorphisms (+896A/G and +1196C/T) may be associated with a significantly increased gastric cancer risk in Caucasian.

## Introduction

Gastric cancer is one of the most common malignancy and the third leading cause of cancer death worldwide, with an estimated 952,000 new cases and 723,000 deaths occurred in 2012 [1]. Although surgical resection is the mainstay of treatment for gastric cancer, overall 5-year relative survival rates for gastric cancer patients remain utterly gloomy with approximately 60% in Asian countries and 20% in western countries [2]. It is well known that gastric cancer is a devastating and multifactorial disease, which derived from extremely complex interactions between genetic and environmental factors [3]. Single Nucleotide Polymorphisms (SNPs) are the most common type of genetic variations and may contribute to an individual's susceptibility to cancer.

Chronic inflammation is well known to play a critical role in initiating and promoting several human cancers including gastric cancer. TLRs activation contributes significantly to the initiation and maintenance of inflammatory response, dysregulation of TLRs signaling may lead to an imbalance state between pro-inflammatory and anti-inflammatory cytokines, and then to an increased susceptibility to chronic inflammatory diseases and cancer [4]. Accumulated evidence has suggested that SNPs located in leucine-rich repeat(LRR) of TLRs may upregulate or downregulate the receptor response ability to bind ligand of pathogens which they normally recognize [5]. Human TLR4 gene is located on chromosome 9q32-q33 and contains four exons. It is highly expressed on monocytes, lymphocytes and splenocytes. After binding with LPS or other ligands and conjunction with CD14 and myeloid differential protein-2 (MD-2), TLR4 could transduce relevant signals that promote transcription of immune-related genes, and thus activate and magnify subsequent immune response including NF-κB and MAPK signal pathways [6], [7]. Many recent studies have demonstrated the critical role of TLR4 in the inflammatory-related immune response to H. Pylori infection in the pathogenesis of gastric cancer. TLR4 polymorphisms allow H. Pylori to elude attack from the host innate immune cell, and to survival in gastric epithelium for a long time, leading to chronic inflammation and causing severe gastritis, hypochlorhydria, gastric atrophy, intestinal metaplasia and dysplasia, which are precancerous lesions of gastric cancer [8]–[12].

Many SNPs in TLR4 have been detected and studied, but two of the most typical SNPs in the fourth exon of coding sequence, +896A/G (ID: rs4986790) and +1196C/T (ID: rs4986791), were explored and reported extensively for the gastric cancer susceptibility [8], [13], [14]. To date, several studies focused on the association of TLR4 (+896A/G and +1196C/T) polymorphisms with precancerous lesions, gastric cancer, across different ethnicities [13]–[16], however, due to the limitations of subjects, the results were inconsistent and controversial. For example, there were two meta-analyses investigating the correlation between TLR4 +896A/G polymorphism and cancer risk, but the results remained partly conflicting [14], [17]. The clinical heterogeneity derived from included studies on diverse histological types of cancers might affect the reliability and stability of the overall results. In addition, the previous meta-analyses did not cover all eligible publications related to gastric cancer and thus decreased the efficacy and probability of detecting the authentic association between TLR4 gene polymorphisms and gastric cancer risk. To clarify and quantify the authentic effect of TLR4 (+896A/G and +1196C/T) polymorphisms on susceptibility to gastric cancer, we performed a meta-analysis including all eligible case-control studies. As far as we know, this was the most comprehensive meta-analysis concerning this subject. All potential genetic models [18] and publication bias detection methods were used in our meta-analysis, which made the results more robust and reliable.

## Materials and Methods

### Identification and eligibility of relevant studies

A systematic search was conducted using the PubMed and EMBase databases (last search was updated on March 26 2014), with the following search details: TLR4 [All Fields] AND (“polymorphism, genetic” [MeSH Terms] OR (“polymorphism” [All Fields] AND “genetic” [All Fields]) OR “genetic polymorphism” [All Fields] OR “polymorphism” [All Fields]) AND (“stomach neoplasms” [MeSH Terms] OR (“stomach” [All Fields] AND “neoplasms” [All Fields]) OR “stomach neoplasms” [All Fields] OR (“gastric” [All Fields] AND “cancer” [All Fields]) OR “gastric cancer” [All Fields]). With the purpose of identifying the extra eligible studies, the references cited in the publications or review articles concerning this topic were manually searched too. Our search was limited to English-language articles. The identified studies in our meta-analysis met the following criteria: (1) studies investigating the association of TLR4 polymorphisms (+896A/G or +1196C/T) with gastric cancer risk; (2) case-control studies and studies included available genotype frequencies; (3) inclusion of comprehensive data to calculate odds ratio (OR) and 95% confidence interval (CI); (4) publications with full-text article. Major criteria for excluding studies were: (1) precancerous lesions or benign tumors; (2) without control subjects; (3) duplicated previous publications; (4) the control subjects did not meet the Hardy-Weinberg Equilibrium (HWE). At last, 10 case-control published studies from PubMed and EMbase were available, including a total of 1888 gastric cancer patients and 3433 control subjects for the TLR4 polymorphisms (+896A/G and +1196C/T). The PRISMA checklist was available as supplementary material, as displayed in Checklist S1.

### Data extraction

Two investigators (QZ and CCW) performed the data extraction independently, and then conducted consensus decision to resolve the disagreements. All study personnel were blinded throughout the meta-analysis. For each study, the following information was collected: the name of first author, publication year, country, ethnicity, design, genotyping method, number of cases and controls, genotype and allele frequencies for cases and controls, and HWE of controls.

### Meta-analysis

The HWE was recalculated in the present meta-analysis according to the HWE principle and formula [19]. And a *P*-value more than 0.05 was considered to meet HWE. Meta-analysis for TLR4 polymorphisms was performed by using the software Stata 12.0 (Stata Corporation, College Station, TX, USA). The association of the TLR4 polymorphisms (+896A/G and +1196C/T) with risk of gastric cancer was estimated by odds ratios (OR) and 95% confidence intervals (CI). The significance of pooled OR was measured by the Z-test, and statistical significance was determined as a 2-sided *P*-value less than 0.05. We measured the association of allele G and allele T (additive model) with gastric cancer risk, and made comparisons with heterozygous model (AG vs. AA) and (CT vs. CC). Heterogeneity assumption was conducted with a χ^2^-based Q-test. If the *P*-value>0.05 for Q-test, thus demonstrating that all studies were lack of heterogeneity, then fixed effect model was adopted to merge studies (the Mantel-Haenszel method) [20], or else the random effect model (the DerSimonian and Laird method) was used [21]. Subgroup meta-analyses according to different races (Caucasian and non-Caucasian) have been conducted based on these genetic models. In addition, sensitivity analysis was conducted to evaluate stability of the results by excluding one study at a time, the pooled ORs were recalculated to assess the altered effect of individual study on the overall results. Furthermore, potential publication bias was diagnosed and measured by using the Begg and Mazumdar rank correlation test [22] and Funnel plots and Egger's regression test [23].

## Results

### Characteristics of eligible studies

**Figure 1:**
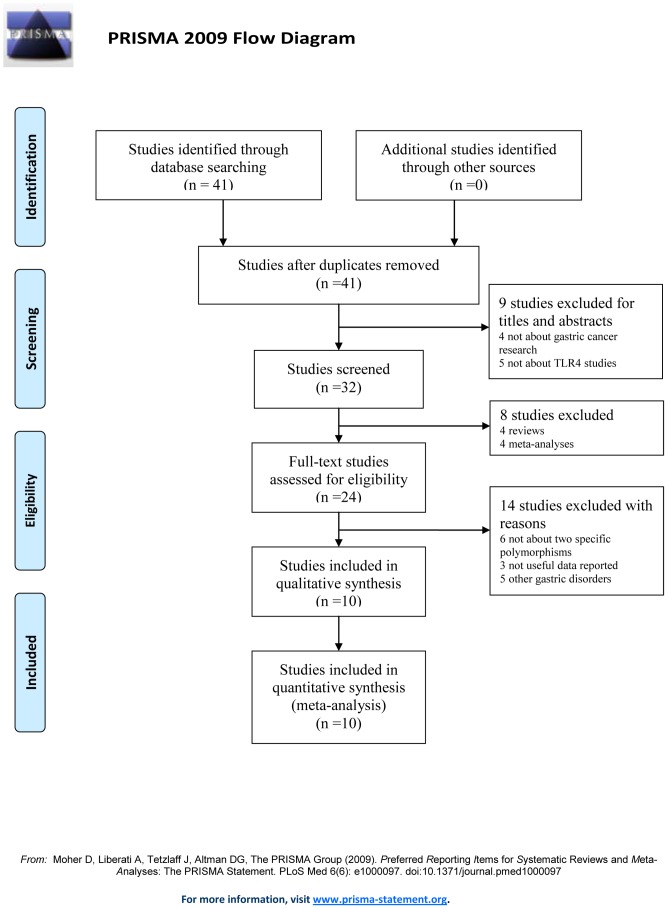
Flow diagram of included and excluded studies.

A total of 41 studies were identified by using different combinations of MeSH terms on PubMed and EMbase. According to the inclusion and exclusion criteria, 10 case-control studies that consisted of a total of 1888 gastric cancer patients and 3433 control subjects were included in this meta-analysis [11], [16], [23]–[30]. Literature search strategy and included or excluded studies were presented in Figure 1. Detailed information, such as author name, publication year, region, ethnicity, design, genotyping method, numbers about cases and controls were summarized in Table 1. The publication year of eligible studies ranged from 2007 to 2014. Two studies adopted hospital-based control, while the other eight studies adopted population-based control. All cases diagnosed with gastric cancer were also validated by pathological examination. All of them adopted blood samples for genotyping. All quality scores of selected articles were higher than 25 (moderate-high quality) [31]. The results of HWE test in the control population and genotype frequencies of TLR4 +896A/G and +1196C/T polymorphisms were recalculated and extracted from all eligible publications, and were shown in Table 2. All the eligible studies met the HWE (all *P*>0.05).

**Table 1:** Characteristics of studies included in the meta-analysis.

Study name	Region	Ethnicity	Design	Genotyping	Case	Control
TLR4(+896A/G,rs4986790)						
Santini(2008)	Italy	Caucasian	PB	PCR-RFLP	171	151
Rigoli(2010)	Italy	Caucasian	PB	PCR-RFLP	60	87
Trejo-de la(2008)	Mexico	Mexican	HB	PCR-sequencing	38	144
de Oliveira(2012)	Brazil	Caucasian	PB	PCR-RFLP	174	225
Hold(2007)a	USA	Caucasian	PB	PCR-RFLP	307	211
Hold(2007)b	Poland	Caucasian	PB	PCR-RFLP	312	419
Garza-Gonzalez(2007)	Mexico	Mexican	HB	PCR-RFLP	78	189
Companioni(2014)	Italy	Caucasian	PB	PCR-sequencing	361	1270
de Oliveira(2013)	Brazil	Caucasian	PB	PCR-RFLP	200	240
Qadri(2013)	India	Asian	PB	PCR-RFLP	130	200
Kutikhin(2014)	Russia	Caucasian	PB	Taqman	57	297
TLR4(+1196C/T,rs4986791)						
Garza-Gonzalez(2007)	Mexico	Mexican	HB	PCR-RFLP	78	156
Santini(2008)	Italy	Caucasian	PB	PCR-RFLP	171	151
Rigoli(2010)	Italy	Caucasian	PB	PCR-RFLP	70	87
Trejo-de la(2008)	Mexico	Mexican	HB	PCR-sequencing	61	202
de Oliveira(2012)	Brazil	Caucasian	PB	PCR-RFLP	174	225
Companioni(2014)	Italy	Caucasian	PB	Taqman	354	1263
de Oliveira(2013)	Brazil	Caucasian	PB	PCR-RFLP	200	240
Qadri(2013)	India	Asian	PB	PCR-RFLP	130	200
Kutikhin(2014)	Russia	Caucasian	PB	Taqman	66	300

HB: hospital based, PB: population based, PCR-RFLP: polymerase chain reaction-restriction fragment length polymorphism.

**Table 2:** The distribution of the TLR4 +896A/G and +1196C/T polymorphisms in gastric cancer.

	Case(n)					Control(n)					
Study name	AA	AG	GG	A	G	AA	AG	GG	A	G	P of HWE
TLR4(+896A/G, rs4986790)											
Santini(2008)	159	11	1	329	13	140	11	0	291	11	0.64
Rigoli(2010)	42	18	0	102	18	80	7	0	167	7	0.7
Trejo-de la(2008)	34	4	0	72	4	138	6	0	282	6	0.18
de Oliveira(2012)	154	20	0	328	20	215	10	0	440	10	0.7
Hold(2007)a	266	38	3	570	44	196	16	1	408	18	0.29
Hold(2007)b	258	51	3	567	57	387	31	1	805	33	0.65
Garza-Gonzalez(2007)	72	6	0	150	6	175	14	0	364	14	0.6
Companioni(2014)	316	45	0	677	45	###	133	3	###	139	0.66
de Oliveira(2013)	174	26	0	374	26	224	16	0	464	16	0.59
Qadri(2013)	107	23	0	237	23	169	31	0	369	31	0.23
Kutikhin(2014)	46	11	0	103	11	258	39	0	555	39	0.23
	Case(n)					Control(n)					
TLR4(+1196C/T,rs4986791)	CC	CT	TT	C	T	CC	CT	TT	C	T	
Garza-Gonzalez(2007)	77	1	0	155	1	179	10	0	368	10	0.71
Santini(2008)	155	15	1	325	17	147	4	0	298	4	0.87
Rigoli(2010)	57	13	0	127	13	81	6	0	168	6	0.74
Trejo-de la(2008)	57	4	0	118	4	193	9	0	395	9	0.75
de Oliveira(2012)	165	9	0	339	9	219	6	0	444	6	0.84
Companioni(2014)	309	45	0	663	45	###	134	5	###	144	0.64
de Oliveira(2013)	191	9	0	391	9	234	6	0	474	6	0.84
Qadri(2013)	114	16	0	244	16	182	18	0	382	18	0.51
Kutikhin(2014)	55	11	0	121	11	255	45	0	555	45	0.16

HWE: Hardy-Weinberg Equilibrium.

### Quantitative data synthesis

**Figure 2:**
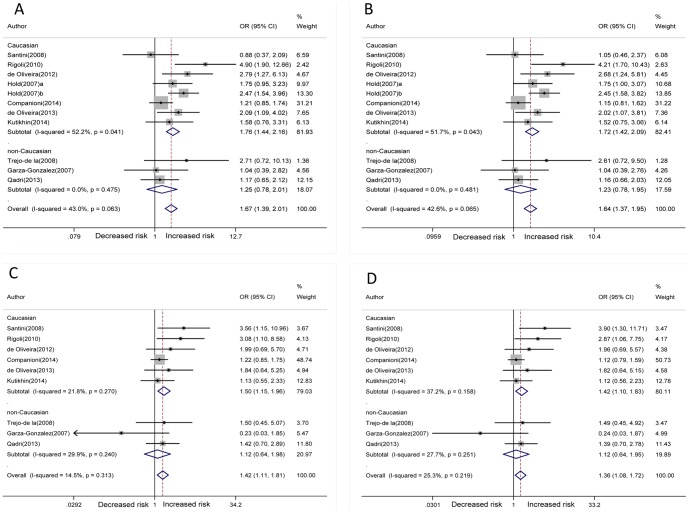
Forest plots showing the association of the TLR4 +896A/G and +1196C/T polymorphisms with risk of gastric cancer. A. AG vs. AA; B. G vs. A; C. CT vs. CC; D. T vs. C.

**Figure 3:**
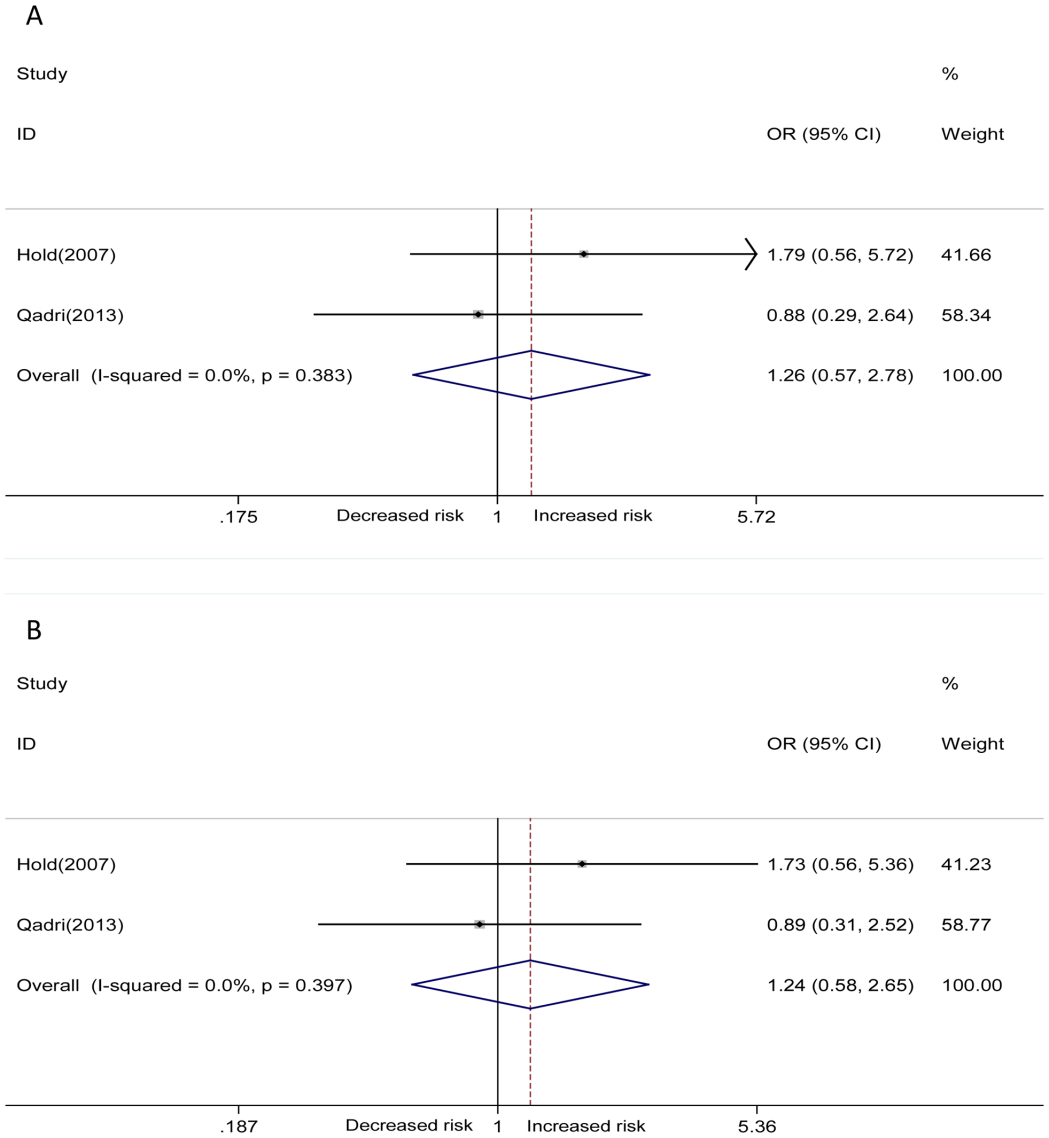
Forest plots showing the association of the TLR4 +896A/G polymorphism with H. Pylori infection in gastric cancer patients. A. AG vs. AA, B. G vs. A.

The overall frequency of G allele in TLR4 +896A/G polymorphism was 7% in cases and 5% in controls. The frequency of the T allele in TLR4 +1196C/T polymorphism was 5% in cases and 4% in controls. For TLR4 +896A/G polymorphism, individuals carrying the variant AG genotype had a significantly increased gastric cancer risk compared with the AA genotype (heterozygous model) (AG vs. AA: OR = 1.67, 95%CI = 1.39–2.01, *P* = 0.000) (Figure 2A). The significance also have been detected in the additive model for the comparison of G allele with A allele (G vs. A: OR = 1.64, 95%CI = 1.37–1.95, *P* = 0.000) (Figure 2B). Moreover, for TLR4 +1196C/T polymorphism, an increased gastric cancer risk was found for the comparison of CT with CC genotype (heterozygous model) (CT vs. CC: OR = 1.42, 95%CI = 1.11–1.81, *P* = 0.005) (Figure 2C), as well as for the comparison of T allele with C allele (additive model) (T vs. C: OR = 1.36, 95%CI = 1.08–1.72, *P* = 0.010) (Figure 2D). Besides, subgroup meta-analyses according to different races (Caucasian and non-Caucasian) have been conducted based on these genetic models. We found significantly positive correlations between the two selected TLR4 polymorphisms and increased risks of gastric cancer in Caucasians, but not in non-Caucasian. The significant association of dominant model (AG+GG vs. AA: OR = 1.68, 95%CI = 1.40–2.02, *P* = 0.000 and CT+TT vs. CC: OR = 1.40, 95%CI = 1.10–1.78, *P* = 0.006) with gastric cancer risk was also observed. In all genetic comparisons, fixed effect model (Mantel-Haenszel method) was used as a result of no obvious heterogeneity (Q-test: *P*>0.05, I^2^<50%) (Table 3). However, there was no significant difference in homozygous model (GG vs. AA and TT vs.CC) or recessive model (GG vs. AG+AA and TT vs. CT+TT). There were two studies investigating the association between TLR4 +896A/G polymorphism and H. Pylori infection in gastric cancer patients [26], [29], and one study for TLR4 +1196C/T polymorphism [29]. However, no significant association was observed in the overall results, TLR4 +896A/G polymorphism did not increase the risk of H. Pylori infection in gastric cancer patients (Figure 3A: AG vs. AA, Figure 3B: G vs. A).

**Table 3:** The results of Q-test in all genetic models for TLR4 +896A/G and +1196C/T polymorphisms in gastric cancer.

Genotype	Q-value	I-squared(%)	P-value	OR(95%CI)	Pooled P-value
TLR4 +896A/G					
AG vs. AA	17.55	43	0.063	1.67(1.39–2.01)	0
GG+AG vs. AA	18.02	44.5	0.055	1.68(1.40–2.02)	0
G vs. A	17.43	42.6	0.065	1.64(1.37–1.95)	0
TLR4 +1196C/T					
CT vs. CC	9.36	14.5	0.313	1.42(1.11–1.81)	0.005
TT+CT vs. CC	10.13	21	0.256	1.40(1.10–1.78)	0.006
T vs. C	10.71	25.3	0.219	1.36(1.08–1.72)	0.01

### Sensitivity analysis

**Figure 4:**
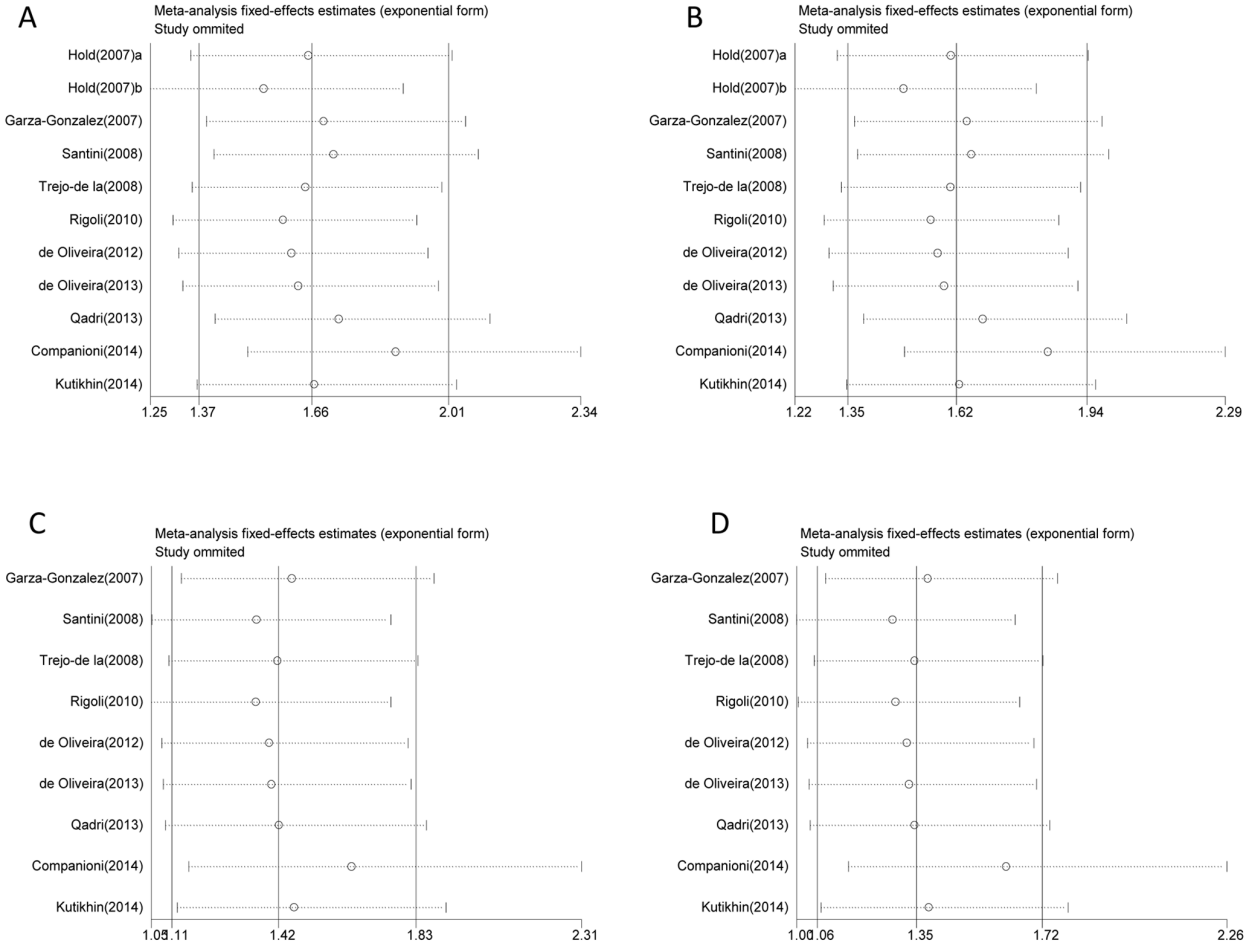
Sensitivity analysis for heterogeneity. A. AG vs. AA; B. G vs. A; C. CT vs. CC; D. T vs. C.

We conducted sensitivity analysis to evaluate the root of heterogeneity. There was no heterogeneity in heterozygous, dominant models and additive models in TLR4 +896A/G and TLR4 +1196C/T polymorphisms. Besides, no single study qualitatively affected the pooled OR, as demonstrated by sensitivity analysis (Figure 4), which suggested that the results of this meta-analysis are reliable and stable. However, there was significant heterogeneity in homozygous and recessive model because that the homozygous genotype GG and TT in many studies were absent.

### Publication bias

**Figure 5:**
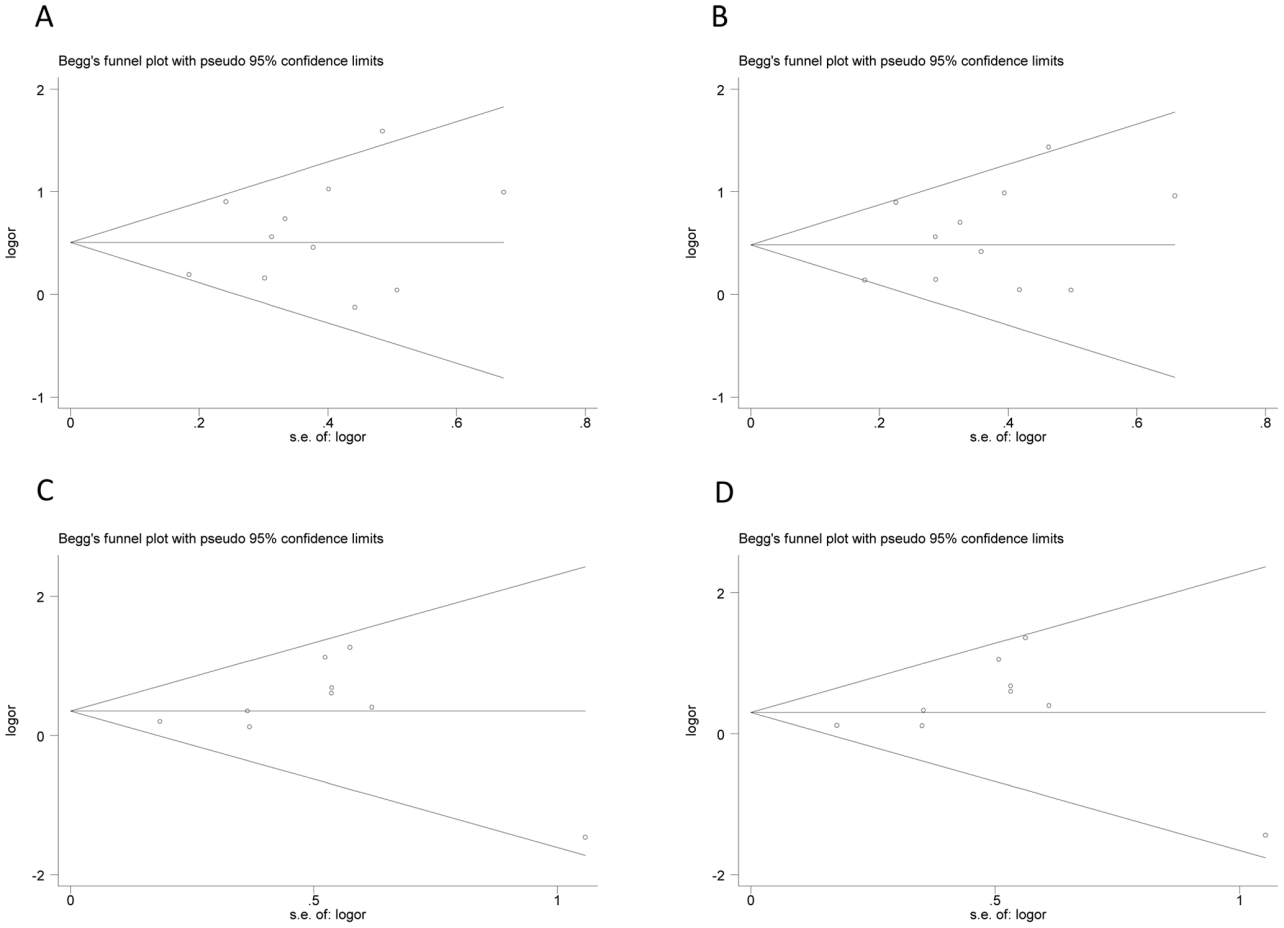
Begg's funnel plots for publication bias test. A. AG vs. AA; B. G vs. A; C. CT vs. CC; D. T vs. C.

**Figure 6:**
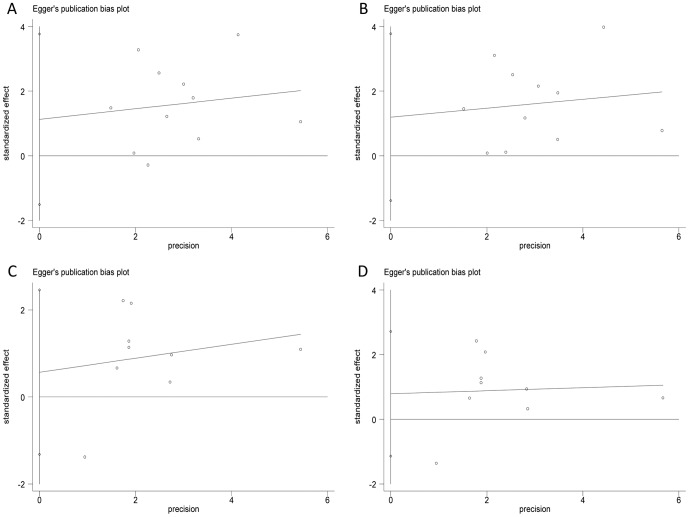
Egger's linear regression plots for publication bias test. A. AG vs. AA; B. G vs. A; C. CT vs. CC; D. T vs. C.

Publication bias within each study might not represent all studies. We performed Begg's funnel plot and Egger's linear regression test to assess the publication bias of all included studies. As demonstrated in Figure 5, the funnel plots did not display any evidence of obvious asymmetry under the heterozygous and additive models (Figure 5A. AG vs. AA: z = 0.78, *P* = 0.436; Figure 5B. G vs. A: z = 0.78, *P* = 0.436; Figure 5C. CT vs. CC: z = 0.52, *P* = 0.602; Figure 5D. T vs. C: z = 0.730, *P* = 0.466). Egger's test also suggested that there was no obvious statistical publication bias under the heterozygous and additive models (Figure 6A. AG vs. AA: t = 0.97, *P* = 0.358; Figure 6B. G vs. A: t = 1.05, *P* = 0.322; Figure 6C. CT vs. CC: t = 0.71, *P* = 0.501; Figure 6D. T vs. C: t = 0.97, *P* = 0.365).

## Discussion

Extensive genetic and epidemiological evidence indicates that chronic inflammation imposes great risk for the development and progression of many kinds of cancers [32], [33], especially for gastric cancer [34], [35]. TLR4 plays a pivotal role in innate immunity defending the body against pathogenic organisms. The recognition of TLR4 by its ligand results in a cascade reaction including the IL-1R family activation and followed by the activation of NF-κB [36], which is a key mediator of inflammation-induced gastric cancer development [7], [37]. However, the function of TLR4 at the protein level will be impaired by SNPs in TLR4 gene, subsequently leading to an altered susceptibility to chronic inflammatory diseases and cancers [38], [39]. The causal link between H. Pylori and gastric cancer has been confirmed. Recent studies have revealed that SNPs within TLR4 is significantly associated with gastric chronic-inflammation and HP infection related mucosa lesions [12], [40]–[42]. Several SNPs within TLR4 have been identified, and the two commonest (+896A/G and +1196C/T) of them that were founded in the fourth exon of coding sequence affect the stability of TLR4 extracellular domain and result in adenine-guanine (A–G) and cytosine-thymine (C–T) exchanges, respectively, and thus finally cause amino acid substitutions: glycine for aspartic at position 299 and isoleucine for threonine at position 399 [43], [44]. Wild-type TLR4 possesses a particular area with negative charge at position 299, which is absent in +896A/G polymorphism, and thus such polymorphism enhances the rotational freedom and angle of the peptide bond in the LPS/MD-2 complex binding area [45]. Consequently, the interaction of TLR4 with LPS may be modulated by the altered rotation and charge, which results in the functional peculiarities of target cells with +896A/G polymorphism [46]. As opposed to +896A/G polymorphism, the branched side chain is conserved in +1196C/T polymorphism, however, the increased three-dimensional bulk in this region, probably preventing ligands or cofactors from docking [45]. Both +896A/G and +1196C/T TLR4 polymorphisms may result in the loss of docking, which cannot be precluded by the remaining functional interactions, and it is verified by the fact that the targeted doubly mutated TLR4 molecule permanently responds more poorly to the stimulation of ligand than TLR4 molecules that possess either the +896A/G or +1196C/T mutation [45]. Individuals with TLR4 polymorphisms possess an increased susceptibility to severe gastric inflammation, hypochlorhydria, gastric atrophy and subsequent development of intestinal metaplasia [8], [10], which the latter two are considered as the most critical precancerous pathological changes associated with gastric cancer.

Although the important role of TLR4 polymorphisms in the development of gastric cancer has been reported in recent years, the results are inconsistent. Garza-Gonzalez et al reported that there was no correlation between TLR4 polymorphisms and gastric cancer in Mexican ethnicity [27], however, Trejo-de la et al insisted that both SNPs (+896A/G and +1196C/T) within TLR4 had an increased susceptibility to gastro-duodenal diseases including duodenal ulcer and gastric cancer [24]. Other studies suggested that only one of these two polymorphisms (either +1196C/T or +896A/G) had association with an increased risk of precancerous lesions and gastric cancer [4], [16]. Hold et al indicated that TLR4 +896A/G polymorphism was associated with increased susceptibility to gastric atrophy and subsequent gastric carcinogenesis [26]. Recently, several studies have shown that both +896A/G and +1196C/T SNPs were related to increased gastric cancer risk [11], [29], [30], while de Oliveira et al has shown not +1196C/T but +896A/G to be associated with such an increased risk in a Brazilian population [25]. Consequently, we performed a comprehensive meta-analysis on the correlation between the TLR4 polymorphisms (+896A/G and +1196C/T) and gastric cancer risk. As far as we know, this was the most comprehensive and updated meta-analysis investigating the association between TLR4 polymorphisms and gastric cancer risk. Because the studies involving in our meta-analysis completely met all the screening criteria, the results from these pooled studies were more powerful than those from single study. After searching the PubMed and EMbase, a total of 10 case-control studies were available including 1888 gastric cancer patients and 3433 control subjects from 2007 to 2014. Consistent with the conclusions resulted from the mentioned studies, our results demonstrate that both +896A/G and +1196C/T TLR4 polymorphisms are associated with a significantly increased gastric cancer, especially for genotype model (heterozygous: AG vs. AA, dominant: GG+AG vs. AA, heterozygous: CT vs. CC, dominant: CT+TT vs. CC) and additive model (G vs. A and T vs. C). Therefore, these two polymorphisms should be regarded as the most significant polymorphisms in TLR4. In the overall +896A/G and +1196C/T meta-analyses, we demonstrated that the minor alleles could obviously increase the risk of gastric cancer in comparison with major alleles, showing that these two genetic variants may critically modify the susceptibility to gastric cancer. In the subgroup meta-analyses, we found significantly positive correlations between the two selected TLR4 polymorphisms and increased risks of gastric cancer in Caucasians, but not in non-Caucasian. The discrepancy between the two races may be attributed to the differences of life styles and diverse environmental exposure factors of the different ethnic populations.

However, there are several limitations of the current meta-analysis, which must be taken into consideration. Because the homozygous genotypes of TLR4 gene (GG and TT) were almost completely absent in the population studied, homozygous and recessive models were absent in the present study. To reconfirm this result, more cases about comprehensive population (Western countries and Asian countries) should be included in the study on the association of TLR4 SNPs with gastric cancer. In addition, articles in other languages (not English) were excluded, which may deviate the results. Besides, subgroup analysis according to gastric cancer site (cardia and noncardia) and histological type, and risk factor analysis for different precancerous lesions according to its type, could provide us more valuable information, but the sample size and individual original data limitations hinder a statistically significant analysis. Finally, TLRs induce the expression of pro-inflammatory genes on gastric mucosa, and the interactions between environmental carcinogens (biologic, chemical and physical stimulations) and host cells, that could be used to elucidate the mechanism by which TLR4 +896A/G and +1196C/T polymorphisms increase risk of developing gastric cancer. More original data need to be obtained to interpret the gene-environment interactions and subsequent gastric carcinogenesis.

In conclusion, this meta-analysis demonstrates that TLR4 +896A/G and +1196C/T polymorphisms probably increase the susceptibility to gastric cancer mainly in Caucasians, while the susceptibility to gastric cancer in Chinese and other Asian population need to be proved in future large scale studies. Although genome-wide association studies (GWAS) are important procedures for the discovery of genetic variations and the reliability of meta-analysis, there are no available previous GWAS on this subject, thus, we have performed sensitivity analysis and publication bias test to avoid such potential discrepancy in this meta-analysis, which suggests no obvious selection bias and confirms the reliability and stability of our results. Since gastric cancer is a multifactorial and multistep disease, it is important to perform well-design and large scale studies, including comprehensive individual data, homogenous patients and underlying source population based controls, standardized genotyping methods to thoroughly reveal the association of TLR4 polymorphisms with gastric cancer risk.

## Supporting Information

Checklist S1 **PRISMA meta-analysis checklist.**
(DOCX)

## References

[1] WHO: World Health Organization: stomach cancer (2014) estimated incidence, mortality and prevalence worldwide in 2012. Available: http://globocan.iarc.fr/Pages/fact_sheets_cancer.aspx. Accessed: 2014 Mar 26.

[2] Kamangar F, Dores GM, Anderson WF (2006) Patterns of cancer incidence, mortality, and prevalence across five continents: defining priorities to reduce cancer disparities in different geographic regions of the world. J Clin Oncol 24: 2137–2150. 16682732 10.1200/JCO.2005.05.2308

[3] Pharoah PD, Dunning AM, Ponder BA, Easton DF (2004) Association studies for finding cancer-susceptibility genetic variants. Nat Rev Cancer 4: 850–860. 15516958 10.1038/nrc1476

[4] Achyut BR, Ghoshal UC, Moorchung N, Mittal B (2007) Association of Toll-like receptor-4 (Asp299Gly and Thr399Ileu) gene polymorphisms with gastritis and precancerous lesions. Hum Immunol 68: 901–907. 18082569 10.1016/j.humimm.2007.10.006

[5] Bell JK, Mullen GE, Leifer CA, Mazzoni A, Davies DR, et al (2003) Leucine-rich repeats and pathogen recognition in Toll-like receptors. Trends Immunol 24: 528–533. 14552836 10.1016/s1471-4906(03)00242-4

[6] Takeda K, Akira S (2005) Toll-like receptors in innate immunity. Int Immunol 17: 1–14. 15585605 10.1093/intimm/dxh186

[7] He C, Chen M, Liu J, Yuan Y (2014) Host genetic factors respond to pathogenic step-specific virulence factors of Helicobacter pylori in gastric carcinogenesis. Mutat Res 759C: 14–26. 10.1016/j.mrrev.2013.09.002 24076409

[8] El-Omar EM, Ng MT, Hold GL (2008) Polymorphisms in Toll-like receptor genes and risk of cancer. Oncogene 27: 244–252. 18176606 10.1038/sj.onc.1210912

[9] Hishida A, Matsuo K, Goto Y, Hamajima N (2010) Genetic predisposition to Helicobacter pylori-induced gastric precancerous conditions. World J Gastrointest Oncol 2: 369–379. 21160888 10.4251/wjgo.v2.i10.369 PMC2999673

[10] Fan YF, Wu YM, Liu H, Yu Y, Jiang YY, et al (2014) TLR4 polymorphisms associated with developing gastric pre-cancer lesions in a Chinese Han population. Hum Immunol 75: 176–181. 24269697 10.1016/j.humimm.2013.11.002

[11] Companioni O, Bonet C, Munoz X, Weiderpass E, Panico S, et al (2014) Polymorphisms of Helicobacter pylori signaling pathway genes and gastric cancer risk in the European Prospective Investigation into Cancer-Eurgast cohort. Int J Cancer 134: 92–101. 23824692 10.1002/ijc.28357

[12] Kato I, Canzian F, Plummer M, Franceschi S, van Doorn LJ, et al (2007) Polymorphisms in genes related to bacterial lipopolysaccharide/peptidoglycan signaling and gastric precancerous lesions in a population at high risk for gastric cancer. Dig Dis Sci 52: 254–261. 17171451 10.1007/s10620-006-9303-1

[13] Zou TH, Wang ZH, Fang JY (2013) Positive association between Toll-like receptor 4 gene +896A/G polymorphism and susceptibility to gastric carcinogenesis: a meta-analysis. Tumour Biol 34: 2441–2450. 23592020 10.1007/s13277-013-0795-y

[14] Zhang K, Zhou B, Wang Y, Rao L, Zhang L (2013) The TLR4 gene polymorphisms and susceptibility to cancer: a systematic review and meta-analysis. Eur J Cancer 49: 946–954. 23084080 10.1016/j.ejca.2012.09.022

[15] Chen J, Hu S, Liang S, Chen Q, Yang Q, et al (2013) Associations between the four toll-like receptor polymorphisms and the risk of gastric cancer: a meta-analysis. Cancer Biother Radiopharm 28: 674–681. 24007538 10.1089/cbr.2012.1395

[16] Santini D, Angeletti S, Ruzzo A, Dicuonzo G, Galluzzo S, et al (2008) Toll-like receptor 4 Asp299Gly and Thr399Ile polymorphisms in gastric cancer of intestinal and diffuse histotypes. Clin Exp Immunol 154: 360–364. 18826495 10.1111/j.1365-2249.2008.03776.x PMC2633233

[17] Jing JJ, Li M, Yuan Y (2012) Toll-like receptor 4 Asp299Gly and Thr399Ile polymorphisms in cancer: a meta-analysis. Gene 499: 237–242. 22441122 10.1016/j.gene.2012.03.045

[18] Zhu B, Wu X, Zhi X, Liu L, Zheng Q, et al (2014) Methylenetetrahydrofolate Reductase C677T Polymorphism and Type 2 Diabetes Mellitus in Chinese Population: A Meta-Analysis of 29 Case-Control Studies. PLoS One 9: e102443. 25047451 10.1371/journal.pone.0102443 PMC4105552

[19] Ward R, Carroll RJ (2014) Testing Hardy-Weinberg equilibrium with a simple root-mean-square statistic. Biostatistics 15: 74–86. 23975799 10.1093/biostatistics/kxt028

[20] Mantel N, Haenszel W (1959) Statistical aspects of the analysis of data from retrospective studies of disease. J Natl Cancer Inst 22: 719–748. 13655060

[21] DerSimonian R, Laird N (1986) Meta-analysis in clinical trials. Control Clin Trials 7: 177–188. 3802833 10.1016/0197-2456(86)90046-2

[22] Begg CB, Mazumdar M (1994) Operating characteristics of a rank correlation test for publication bias. Biometrics 50: 1088–1101. 7786990

[23] Egger M, Davey Smith G, Schneider M, Minder C (1997) Bias in meta-analysis detected by a simple, graphical test. BMJ 315: 629–634. 9310563 10.1136/bmj.315.7109.629 PMC2127453

[24] Trejo-de la OA, Torres J, Perez-Rodriguez M, Camorlinga-Ponce M, Luna LF, et al (2008) TLR4 single-nucleotide polymorphisms alter mucosal cytokine and chemokine patterns in Mexican patients with Helicobacter pylori-associated gastroduodenal diseases. Clin Immunol 129: 333–340. 18755634 10.1016/j.clim.2008.07.009

[25] de Oliveira JG, Silva AE (2012) Polymorphisms of the TLR2 and TLR4 genes are associated with risk of gastric cancer in a Brazilian population. World J Gastroenterol 18: 1235–1242. 22468087 10.3748/wjg.v18.i11.1235 PMC3309913

[26] Hold GL, Rabkin CS, Chow WH, Smith MG, Gammon MD, et al (2007) A functional polymorphism of toll-like receptor 4 gene increases risk of gastric carcinoma and its precursors. Gastroenterology 132: 905–912. 17324405 10.1053/j.gastro.2006.12.026

[27] Garza-Gonzalez E, Bosques-Padilla FJ, Mendoza-Ibarra SI, Flores-Gutierrez JP, Maldonado-Garza HJ, et al (2007) Assessment of the toll-like receptor 4 Asp299Gly, Thr399Ile and interleukin-8 -251 polymorphisms in the risk for the development of distal gastric cancer. BMC Cancer 7: 70. 17462092 10.1186/1471-2407-7-70 PMC1868033

[28] de Oliveira JG, Rossi AF, Nizato DM, Miyasaki K, Silva AE (2013) Profiles of gene polymorphisms in cytokines and Toll-like receptors with higher risk for gastric cancer. Dig Dis Sci 58: 978–988. 23086128 10.1007/s10620-012-2460-5

[29] Qadri Q, Rasool R, Afroze D, Naqash S, Gulzar GM, et al (2013) Study of TLR4 and IL-8 Gene Polymorphisms in H.pylori-Induced Inflammation in Gastric Cancer in an Ethnic Kashmiri Population. Immunol Invest 43: 324–336. 24295404 10.3109/08820139.2013.854378

[30] Kutikhin AG, Yuzhalin AE, Volkov AN, Zhivotovskiy AS, Brusina EB (2014) Correlation between genetic polymorphisms within IL-1B and TLR4 genes and cancer risk in a Russian population: a case-control study. Tumour Biol 35: 4821–4830. 24446182 10.1007/s13277-014-1633-6

[31] da Costa BR, Cevallos M, Altman DG, Rutjes AW, Egger M (2011) Uses and misuses of the STROBE statement: bibliographic study. BMJ Open 1: e000048. 10.1136/bmjopen-2010-000048 PMC3191404 22021739

[32] Cole SW (2009) Chronic inflammation and breast cancer recurrence. J Clin Oncol 27: 3418–3419. 19470918 10.1200/JCO.2009.21.9782 PMC4828958

[33] Kazma R, Mefford JA, Cheng I, Plummer SJ, Levin AM, et al (2012) Association of the innate immunity and inflammation pathway with advanced prostate cancer risk. PLoS One 7: e51680. 23272139 10.1371/journal.pone.0051680 PMC3522730

[34] Bornschein J, Malfertheiner P (2011) Gastric carcinogenesis. Langenbecks Arch Surg 396: 729–742. 21611816 10.1007/s00423-011-0810-y

[35] Wang F, Meng W, Wang B, Qiao L (2014) Helicobacter pylori-induced gastric inflammation and gastric cancer. Cancer Lett 345: 196–202. 23981572 10.1016/j.canlet.2013.08.016

[36] Aderem A, Ulevitch RJ (2000) Toll-like receptors in the induction of the innate immune response. Nature 406: 782–787. 10963608 10.1038/35021228

[37] Huang B, Zhao J, Li H, He KL, Chen Y, et al (2005) Toll-like receptors on tumor cells facilitate evasion of immune surveillance. Cancer Res 65: 5009–5014. 15958541 10.1158/0008-5472.CAN-05-0784

[38] Armant MA, Fenton MJ (2002) Toll-like receptors: a family of pattern-recognition receptors in mammals. Genome Biol 3: REVIEWS3011. 12186654 10.1186/gb-2002-3-8-reviews3011 PMC139401

[39] Liew FY, Xu D, Brint EK, O'Neill LA (2005) Negative regulation of toll-like receptor-mediated immune responses. Nat Rev Immunol 5: 446–458. 15928677 10.1038/nri1630

[40] Schmausser B, Andrulis M, Endrich S, Lee SK, Josenhans C, et al (2004) Expression and subcellular distribution of toll-like receptors TLR4, TLR5 and TLR9 on the gastric epithelium in Helicobacter pylori infection. Clin Exp Immunol 136: 521–526. 15147355 10.1111/j.1365-2249.2004.02464.x PMC1809056

[41] Hamajima N (2003) Persistent Helicobacter pylori infection and genetic polymorphisms of the host. Nagoya J Med Sci 66: 103–117. 14727687

[42] Bagheri N, Azadegan-Dehkordi F, Sanei H, Taghikhani A, Rahimian G, et al (2014) Associations of a TLR4 single-nucleotide polymorphism with H. pylori associated gastric diseases in iranian patients. Clin Res Hepatol Gastroenterol 38: 366–371. 24508388 10.1016/j.clinre.2013.12.004

[43] Arbour NC, Lorenz E, Schutte BC, Zabner J, Kline JN, et al (2000) TLR4 mutations are associated with endotoxin hyporesponsiveness in humans. Nat Genet 25: 187–191. 10835634 10.1038/76048

[44] Mockenhaupt FP, Cramer JP, Hamann L, Stegemann MS, Eckert J, et al (2006) Toll-like receptor (TLR) polymorphisms in African children: common TLR-4 variants predispose to severe malaria. J Commun Dis 38: 230–245. 17373355

[45] Rallabhandi P, Bell J, Boukhvalova MS, Medvedev A, Lorenz E, et al (2006) Analysis of TLR4 polymorphic variants: new insights into TLR4/MD-2/CD14 stoichiometry, structure, and signaling. J Immunol 177: 322–332. 16785528 10.4049/jimmunol.177.1.322

[46] Ferwerda B, McCall MB, Verheijen K, Kullberg BJ, van der Ven AJ, et al (2008) Functional consequences of toll-like receptor 4 polymorphisms. Mol Med 14: 346–352. 18231573 10.2119/2007-00135.Ferwerda PMC2215765

